# Hemophagocytic lymphohistiocytosis presenting as acute coronary syndrome

**DOI:** 10.4322/acr.2021.285

**Published:** 2021-05-06

**Authors:** Gabriel Melki, Mina Fransawy Alkomos, Sushant Nanavati, Vinod Kumar, Christina Mariyam, Michael Maroules

**Affiliations:** 1 St. Joseph’s University Medical Center, Internal Medicine Department, Paterson, NJ, USA; 2 St. Joseph’s University Medical Center, Pulmonary and Critical Care Department, Paterson, NJ, USA; 3 St. Joseph’s University Medical Center, Hematology and Oncology Department, Paterson, NJ, USA

**Keywords:** Lymphohistiocytosis, Hemophagocytic, Acute Coronary Syndrome, Glucose 6 Phosphate Dehydrogenase Deficiency

## Abstract

Acquired Hemophagocytic Lymphohistiocytosis is a rare and deadly syndrome resulting from an overactive immune system, with uncontrolled activation of macrophages and lymphocytes, hypercytokinemia, and systemic inflammatory response. A 75-year-old male presented with typical anginal pain and was diagnosed with the acute coronary syndrome, which required a percutaneous transluminal coronary angioplasty. Instead of resolving the symptoms, the patient began to exhibit pyrexia and worsening altered sensorium with progressing renal failure, anemia, thrombocytopenia and respiratory failure. This constellation of symptoms caused the patient to require mechanical ventilation and hemodialysis. Upon laboratory analysis, hyperferritinemia provided an indication to the diagnosis of acquired hemophagocytic lymphohistiocytosis. After the initiation of dexamethasone, the patient made a significant recovery and was discharged from the hospital.

## INTRODUCTION

Hemophagocytic Lymphohistiocytosis (HLH) is an underdiagnosed syndrome caused by the overwhelming dysregulation of the immune system, resulting in end-organ damage or even death if untreated. It is described as a hyperinflammatory reaction with uncontrolled, persistent activation of lymphocytes and macrophages engulfing hematopoietic cells, creating a vicious cycle of a cytokine storm.^1^ In 1952, Farquhar and Claireaux reported a case series of two infants presenting with fatal cytopenias, hepatosplenomegaly, and pyrexia, and subsequently termed the condition as *familial hemophagocytic reticulosis*.^2^ Over the years, several cases were published with a similar constellation of symptoms in pediatric and adult populations, though HLH is more common in children and adolescents. Due to its rarity, recognizing and diagnosis HLH continues to be a challenge. HLH is associated with several conditions including malignancy, infection, autoimmune and hereditary diseases. HLH has no predilection with race, sex or age.^1^^,^^3^ We describe the unique case of an elderly male with typical anginal pain who, after undergoing percutaneous coronary intervention, developed multi-organ failure secondary to HLH.

## CASE REPORT

A 75-year-old African American male with a history of Glucose-6-Phosphate Dehydrogenase (G-6-PD) deficiency presented with complaints of retrosternal chest pain. He was admitted for acute coronary syndrome, requiring aspirin, a heparin infusion and atorvastatin. Initial blood work showed hemoglobin 12.3 g/dL (13.5-17.5 g/dl), white blood cells (WBC) 7.2 cells/mm^3^ (4-11 cells/mm^3^) platelets 135 K/mm^3^ (40-799 K/mm^3^) activated partial thromboplastin time (aPTT) 30 seconds (21-35 seconds), Prothrombin time/ International Normalized Ratio (PT-INR) 1.2, troponin-I 0.025 ng/mL (Normal <0.05 ng/ml), blood urea nitrogen (BUN) 11 mg/dl, creatinine 1.13 mg/dl (0.6-1.3 mg/dl), total cholesterol 83 mg/dL, triglyceride 195 mg/dL. EKG showed normal sinus rhythm, normal axis, T wave Inversion In lateral leads (V4-V6), QTC 389 ms unchanged from prior. After a positive nuclear stress test the following day, he underwent a left heart catheterization requiring placement of a drug-eluting stent in the right coronary artery due to 75% mid occlusion, and non-obstructive CAD elsewhere. Aspirin and ticagrelor were initiated post-percutaneous coronary intervention.

Over the next two days, he became increasingly lethargic and progressively obtunded. On Day 4, the patient presented with pyrexia as high as 103 degrees F (38,8 °C) with worsening mental status and hypotension episodes requiring intensive care and mechanical ventilation.

Investigation revealed hemoglobin 7.7 g/dL, WBC 8 cells/mm^3^, platelet count 83 K/mm^3^, BUN 54 mg/dl, creatinine 4.33 mg/dl, alanine aminotransferase (ALT) 37 U/L (7-52 U/L), aspartate aminotransferase (AST) 180 U/L (13-39 U/L), alkaline phosphatase (ALP) 49 IU/L (34-104 IU/L), and LDH 1925 units/l (140-271 units/l). With hospitalization for ACS requiring anticoagulation and his history of G-6-PD, acute hemolysis was suspected, requiring transfusing two units of packed cells. The quantitative G-6-PD level was 8.6 (13-19 U/g Hb). Direct and indirect Coombs tests were negative. There was no evidence of disseminated intravascular hemolysis or thrombotic thrombocytopenic purpura (no schistocytes on peripheral smear). Heparin-Induced Thrombocytopenia (HIT) antibody and serotonin release assay were also negative.

**Figure 1:**
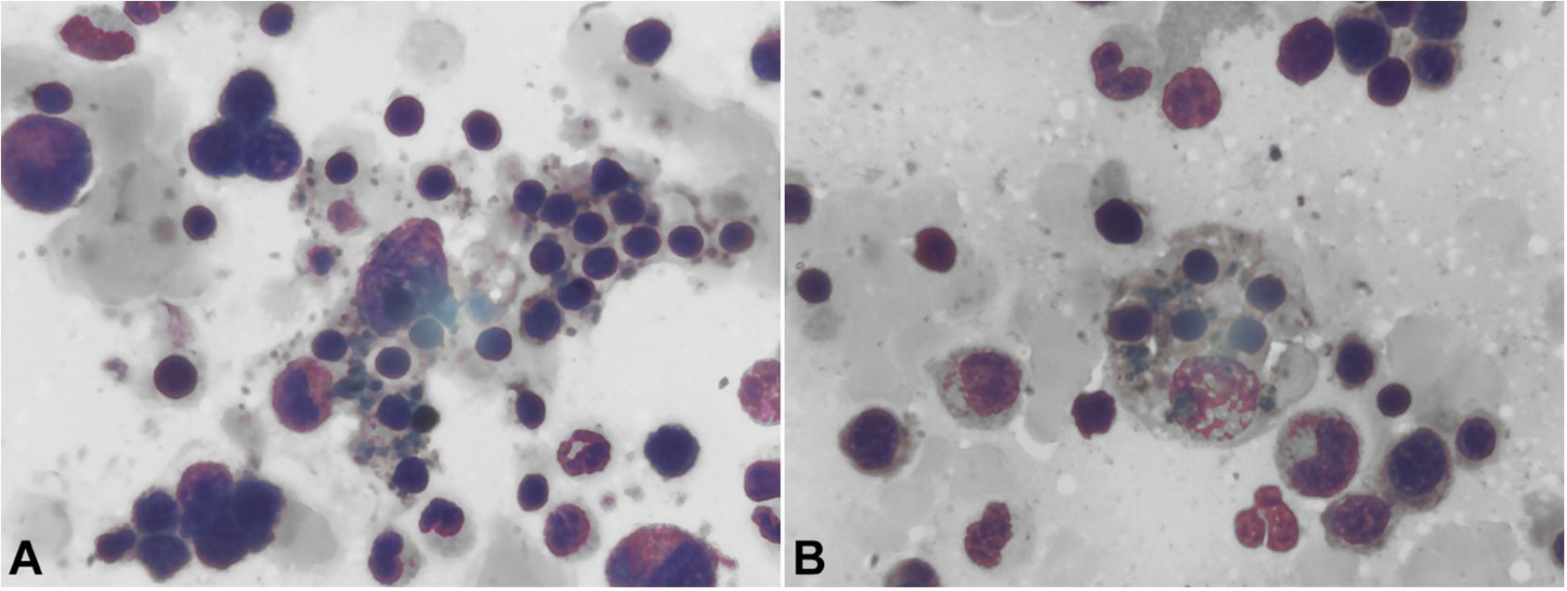
Bone Marrow Biopsy revealing trilineage hematopoiesis with hemophagocytosis.

As progressively worsening creatinine continued, the patient was started on renal replacement therapy (RRT) for acute renal failure. Except for low-grade viremia with Epstein-Barr virus (EBV PCR 800 copies/ mL), Human Immunodeficiency Virus (HIV), hepatitis and respiratory viral panels were negative. Serum procalcitonin and multiple cultures of body fluids were negative for systemic bacterial or fungal infections. While the patient’s fever persisted, despite broad-spectrum antibiotics, a whole-body gallium uptake scan was inconclusive for any localized infection. A ferritin level was ordered as part of routine testing and was found to be elevated at >15,000 ng/mL. This incidental finding proved to be the nexus of this case and prompted further evaluation due to high suspicion for hemophagocytic lymphohistiocytosis (HLH). As part of the HLH workup, the patient underwent a bone marrow biopsy which revealed trilineage hematopoiesis with hemophagocytosis. The following results were obtained: serum triglyceride level of 704 mg/dL, quantitative Fibrinogen of 328 mg/dl (RR; 183-503 mg/dl), and soluble interleukin 2 receptors of 2200 pg/mL (RR; 241-846 U/mL). The patient met five out of eight diagnostic criteria for HLH: fever, bi-lineage peripheral cytopenia, hyperferritinemia, hypertriglyceridemia, elevated IL-2 receptor level, and bone marrow aspirate hemophagocytosis (Figure 1).

After the confirmation of HLH, the patient was initiated on dexamethasone 10 mg intravenous every twelve hours. With the initiation of treatment, many of his symptoms began to lessen: fevers resolved and mentation improved. Ultimately the patient was successfully extubated on day 10. Although he initially required additional packed red cell transfusions, his hemoglobin stabilized while ferritin and triglyceride levels decreased.

The patient continued to improve and was discharged in stable condition with recommendations to continue dexamethasone therapy. At follow-up in the outpatient clinic, he had no relapse episodes on the treatment, and creatinine stabilized requiring no further hemodialysis session.

## DISCUSSION

Cytokine storm induced by the uninhibited release of inflammatory cytokines (interferon-gamma (IFN-γ), interleukin-2 and tumor necrosis factor) may result in diffuse vascular endothelial damage, significant myelosuppression, and considerable mortality in HLH.^4^

This disease may be classified as primary (hereditary or familial HLH) or secondary (acquired HLH). Primary HLH typically manifests in neonates and children, with chromosomal abnormalities influencing the function of natural killer (NK) cells and cytotoxic T-cells.^4^ Pathogenesis of primary HLH originates from the functional impairment of NK cells and cytotoxic T-cells, causing macrophage activation from the amplified cytokines secretion resulting in increasing serum cytokines.^4^

The mechanism of acquired HLH is broadly diverse and poorly understood compared to primary HLH. Since acquired HLH continues to be underdiagnosed, the true prevalence of this disease remains uncertain. In addition, secondary or acquired HLH is associated with numerous conditions, including viral infections, malignancies, autoimmune disorders, and immune deficiency disorders. In terms of malignancy, lymphomas are commonly associated with HLH, and when HLH occurs in autoimmune disorders in particular adult Still’s disease, it is termed macrophage activation syndrome (MAS).^1^^,^^3^

In a review published in 2014 by George,^5^ it was reported that the etiologies associated with HLH are; viral infections (29%), other infections (20%), malignancies (27%), rheumatologic disorders (7%), and immune deficiency syndromes (6%). Meanwhile, a retrospective analysis of 62 adults with HLH showed a majority malignancy (52%) followed by infection (34%), an autoimmune disorder (8%), and idiopathic causes (6%).^6^ In a study published by Rivière et al.,^7^ 162 adult patients with HLH showed consistency with malignancy (60%), infection (25%), and autoimmune disorders (3%). Within infectious etiology, Epstein-Barr virus (EBV) along with herpes simplex virus (HSV)-1 are common triggers, possibly via a synergistic effect leading to acquired HLH.^1^^,^^3^^,^^4^ In the primary infection of EBV, the virus multiplies in B-cells, causing dysregulation and eventual target designation for the cytotoxic T-cells. EBV may also occur in immunocompetent individuals, where it may infect cytotoxic T-cells causing dysregulation and evasion from the immune system. Both of these evasive mechanisms can cause EBV-HLH.^1^^,^^4^^,^^8^

Rare presentations, nonspecific symptoms, symptomatic overlap, and unexplained multiorgan failure require a high index of suspicion to diagnose HLH, which makes this disease particularly evasive of differential diagnoses and thereby particularly fatal. In 1991, the Histiocyte Society proposed a diagnostic criterion for the pediatric population, and this was widely used for adult or acquired HLH by the time it was revised in 2004.^1^^,^^3^^-^^5^^,^^9^ Clinical symptoms and laboratory investigations which are part of diagnostic criteria are directly associated with the pathophysiology of HLH: aggregation of lymphocytes and macrophages causing splenomegaly, elevated interleukin 1 resulting in fever, high tumor necrosis factor yielding hypertriglyceridemia, inflammatory macrophage scavenging causing elevated ferritin, and elevated levels of interferon-gamma and tumor necrosis factor leading to cytopenia.^1^^,^^4^^,^^5^^,^^10^

Treatment should target eliminating the inciting trigger, arresting the inflammatory immune system, and initiating immediate specific or supportive therapy. The Histiocyte Society recommends an 8-week course of corticosteroids, etoposide and cyclosporine A for HLH. Dexamethasone, however, remains the cornerstone to suppress hypercytokinemia, especially in acquired HLH.^3^^-^^7^^,^^9^

Also; ticagrelor may trigger thrombotic thrombocytopenic purpra, but not hemophagocytic lymphohistocytosis although rarely, some drugs (sulfamethoxazole-trimethoprim, vancomycin, phenytoin, phenobarbital, and lamotrigine) may eventually trigger hemophagocytic lymphohistiocytosis.^11^ Hypothetically; HLH in this case has Indeterminate etiology.

To our knowledge, there are no previously reported cases of cardiac manifestations triggering or G-6PD associated HLH in adults. Our case highlights the importance of HLH as a part of differential diagnosis in cases of fever, cytopenias, hyperinflammatory or multiorgan failure; hence prompting diagnosis will initiate early therapy to reduce mortality.
